# Tumor-associated macrophages in colorectal cancer metastasis: molecular insights and translational perspectives

**DOI:** 10.1186/s12967-024-04856-x

**Published:** 2024-01-16

**Authors:** Siyu Hou, Yuanchun Zhao, Jiajia Chen, Yuxin Lin, Xin Qi

**Affiliations:** 1 School of Chemistry and Life Sciences, Suzhou University of Science and Technology, Suzhou, 215011 China; 2 Department of Urology, The First Affiliated Hospital of Soochow University, Suzhou, 215000 China; 3 Center for Systems Biology, Department of Bioinformatics, School of Biology and Basic Medical Sciences, Soochow University, Suzhou, 215123 China

**Keywords:** CRC metastasis, Macrophage, TAM, TME, Personalized therapy

## Abstract

Metastasis is the leading cause of high mortality in colorectal cancer (CRC), which is not only driven by changes occurring within the tumor cells, but is also influenced by the dynamic interaction between cancer cells and components in the tumor microenvironment (TME). Currently, the exploration of TME remodeling and its impact on CRC metastasis has attracted increasing attention owing to its potential to uncover novel therapeutic avenues. Noteworthy, emerging studies suggested that tumor-associated macrophages (TAMs) within the TME played important roles in CRC metastasis by secreting a variety of cytokines, chemokines, growth factors and proteases. Moreover, TAMs are often associated with poor prognosis and drug resistance, making them promising targets for CRC therapy. Given the prognostic and clinical value of TAMs, this review provides an updated overview on the origin, polarization and function of TAMs, and discusses the mechanisms by which TAMs promote the metastatic cascade of CRC. Potential TAM-targeting techniques for personalized theranostics of metastatic CRC are emphasized. Finally, future perspectives and challenges for translational applications of TAMs in CRC development and metastasis are proposed to help develop novel TAM-based strategies for CRC precision medicine and holistic healthcare.

## Introduction

Colorectal cancer (CRC) is the third commonly diagnosed cancer and the second deadliest cancer globally [1]. Metastasis is the primary driver of the high mortality in CRC. Statistically, approximately 20% of patients are present with the initial clinical manifestations of metastatic CRC, and up to 50% of patients will eventually develop distant metastases in the liver, lung or other sites [2]. Notably, CRC metastasis is a highly complex and heterogeneous progression process, posing great challenges to the effective treatment of patients. The 5-year survival rate of CRC patients is between 25% and 58% even after treatment [3]. Therefore, it is urgent to explore the mechanism of CRC metastasis, thereby developing promising treatments for clinical benefits.

CRC metastasis is not only driven by the complex alterations in cancer cells, but also mediated by the dynamic crosstalk between cancer cells and their microenvironment. The tumor microenvironment (TME) is a highly heterogeneous and structured ecosystem comprising tumor cells and diverse non-cancerous cells (e.g., endothelial cells, fibroblasts, stromal cells, adaptive immune cells and innate immune cells), embedded in a dynamic extracellular matrix. Accumulating evidence indicates that the TME plays a critical role in CRC development, progression and metastasis [4, 5]. For example, Shang et al. found that VEGFR2 antibody fused with a mutated form of IFN-α could effectively modulate the TME of CRC, producing anti-tumor and anti-metastatic effects [6]. Wei et al. demonstrated that enhanced ketogenesis could ameliorate the immunosuppressive TME by suppressing KLF5-dependent CXCL12 expression, thereby inhibiting the metastasis of CRC [7]. Therefore, exploration of the mechanisms underlying the relationship between TME remodeling and CRC metastasis have currently attracted considerable attention.

Tumor-associated macrophages (TAMs), as crucial players in the TME, can exhibit remarkable plasticity in response to varying environmental cues and therapeutic interventions [8, 9]. Notably, TAMs have emerged as pivotal contributors, exhibiting a complex and dynamic role in CRC progression, particularly in the context of metastasis. The study by Wei et al. demonstrated that TAMs can promote the migration, invasion, and metastasis of CRC by inducing the epithelial-mesenchymal transition (EMT) process. This is achieved through the regulation of the JAK2/STAT3/miR-506-3p/FoxQ1 axis, which in turn trigger the production of CCL2 to facilitate macrophage recruitment [10]. Liu et al. discovered that TAM-secreted TGF-β can activate HIF1α/TRIB3 signaling pathway, thereby promoting the progression of CRC [11]. Moreover, the crosstalk between TAMs and other immune cells, as well as their interactions with tumor cells and stromal elements, shapes the overall microenvironment conducive to CRC metastasis. Therefore, exploring the potential of TAMs as a therapeutic target for managing CRC metastasis is of great translational importance.

Here, we provide a comprehensive and updated overview of the definition, origin, polarization of TAMs, with a particular focus on their roles in promoting the metastatic cascade of CRC. Furthermore, we discuss the promising therapeutic strategies targeting TAMs for CRC patients. It is hoped that this review will contribute to understand the role of TAMs in CRC metastasis and the clinical implications of TAMs in cancer interventional therapy.

## Origin and polarization of TAMs

### Origins and functions of TAMs

**Fig. 1:**
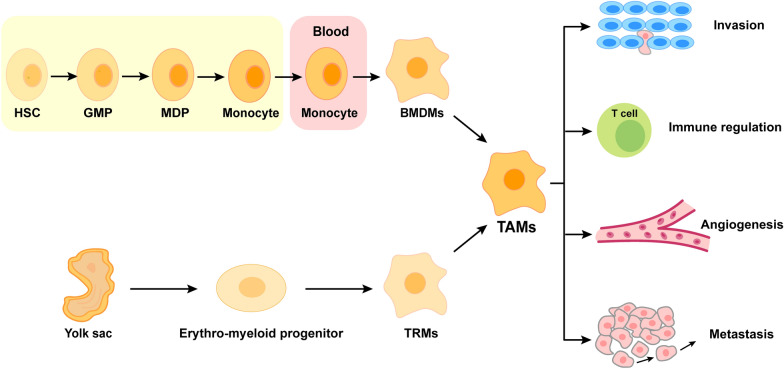
The origin and function of TAMs. As the main source of macrophages, bone marrow-derived monocytes are derived from hematopoietic stem cells (HSCs) that undergo differentiation into granulocyte-macrophage progenitors (GMPs) and subsequently into monocyte-dendritic cell progenitors (MDPs). Monocytes in the blood can be recruited and differentiated into TAM by chemokines (e.g., CCL2, CCL5 and CCL7) or growth factors (e.g., VEGF and PDGF). In addition, yolk sac progenitor cells are another important source of TAMs. Under the stimulation of cytokines (such as IL-6 and IL-8, etc.) and chemokines (such as CCL2 and CXCL8, etc.), TAMs play an important role in tumor invasion, immune regulation, angiogenesis and metastasis. *HSCs *hematopoietic stem cells, *GMPs *granulocyte-macrophage progenitors,* MDPs *monocyte-dendritic cell progenitors, *BMDMs *bone marrow-derived macrophages,* TRMs *tissue-resident macrophages, *TAMs *tumor-associated macrophages

Macrophages are a type of immune cells that play a crucial role in defending the host against pathogens, regulating tissue homeostasis and maintaining tissue architecture. Macrophages infiltrating tumor tissues or present in the TME are defined as TAMs, which can play a pro-tumor role by affecting tumor growth, angiogenesis, metastasis, drug resistance, and immunosuppression [12, 13]. According to the origin and resident location, TAMs are generally developed from two ways: (1) TAMs arise from bone marrow-derived macrophages (BMDMs) in response to tumor growth, which are formed by the differentiation from circulating monocytes that are produced by hematopoietic stem cells (HSCs) in the bone marrow; (2) TAMs originate from tissue-resident macrophages (TRMs) that are established by erythromyeloid progenitor cells in the yolk sac (Fig. 1) [14]. Accordingly, the former are also termed as tumor-induced TAMs, while the latter are also known as tissue-resident TAMs. Increasing evidence shows that bone marrow-derived monocytes can be recruited and differentiated into TAMs by a variety of chemokines and growth factors released by tumor cells and stromal cells in the TME, such as C-C chemokine ligand 2 (CCL2), CCL5, CCL7, C-X-C motif chemokine ligand 8 (CXCL8), CXCL12, granulocyte macrophage colony stimulating factor (GM-CSF), macrophage colony promoting factor (M-CSF), vascular endothelial growth factor (VEGF), and platelet derived growth factor (PDGF) [15, 16]. Comparatively, TRMs migrate into distinct tissues during embryonic development, generating self-renewing populations that persist into adulthood without hematopoietic stem cells (HSCs) involvement [14]. It should be noted that tumor-induced TAMs and tissue-resident TAMs may co-exist in a particular tumor tissue with different dominating roles in the early and later stages of tumor progression [17]. The distinct origins add to the heterogeneity and complexity of the TME, suggesting that the origin of TAMs should be considered in further studies.

### Polarization of TAMs

**Fig. 2:**
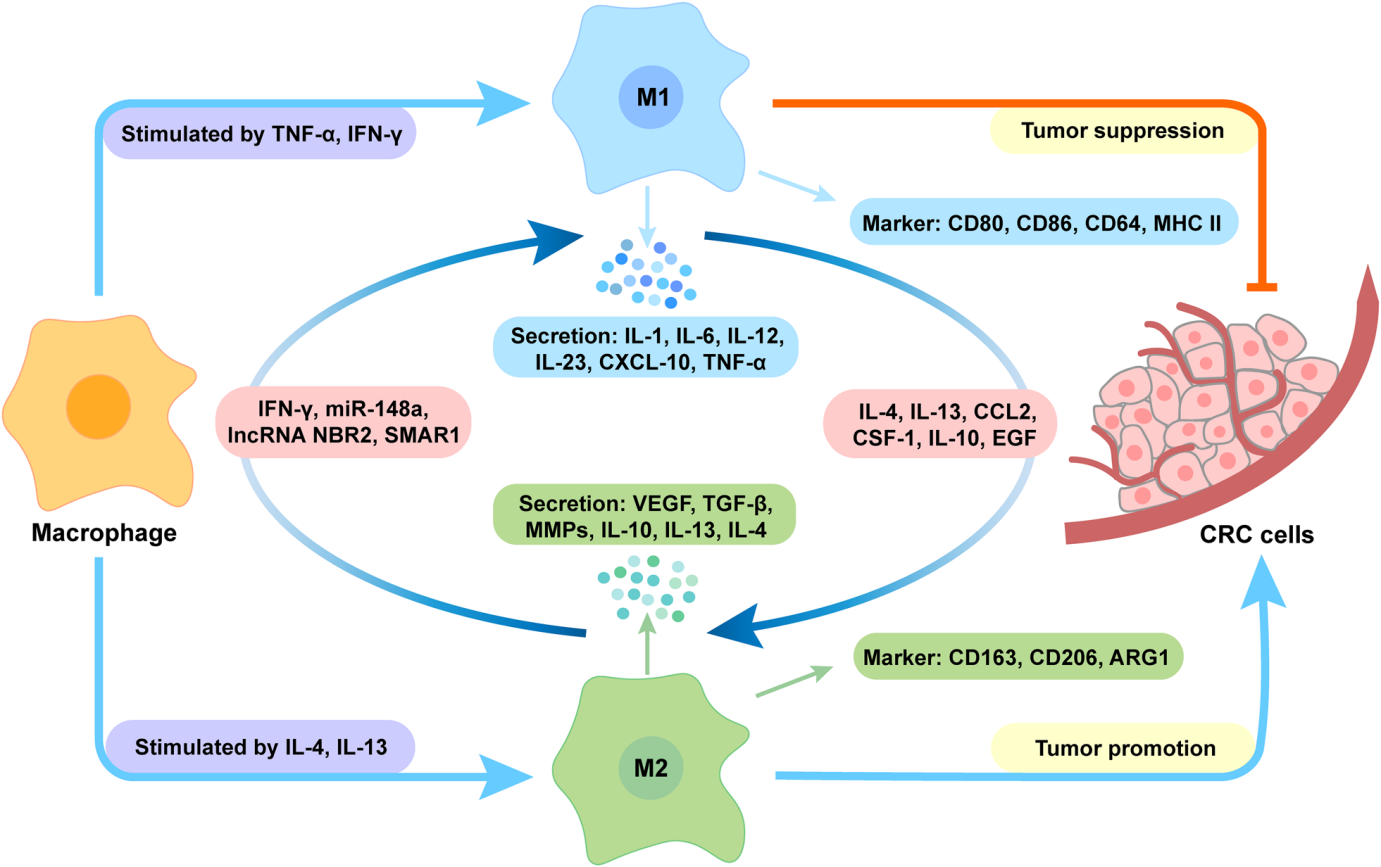
Polarization of TAM and their biological properties. Stimulated by cytokines (such as TNF-α, IFN-γ, etc.), M1-type macrophages can secrete cytokines (such as IL-1, IL-6, etc.), express CD64, CD80, CD86, and MHC II, and exhibit anti-tumor effects. Comparatively, stimulated by cytokines (such as IL-4, IL-13, etc.), M2-type macrophages can secrete IL-10 and TGF-β, express CD206, CD163, ARG1, and exhibit pro-tumor effects. Under the stimulation of different molecules (such as SMAR1, etc.) and factors (such as CSF-1, CCL2, etc.), M1-type macrophages and M2-type macrophages can transform into each other. *TNF-α *tumor necrosis factor-α, *IFN-γ *interferon-γ, *IL-4 *interleukin-4, *IL-13 *interleukin-13, *IL-1* interleukin-1, *IL-6 *interleukin-6, *IL-12 *interleukin-12, *IL-23 * interleukin-23, *IL-10 *interleukin-10, *CXCL-10 *C-X-C motif chemokine ligand 10, *VEGF *vascular endothelial growth factor, *TGF-β *transforming growth factor-β, *MMPs *matrix metalloproteinases, *CCL2 *C-C chemokine ligand 2, *CSF-1 *colony stimulating factor-1, *EGF *epidermal growth factor

TAMs are highly plastic cells capable of differentiating into two distinct phenotypes: anti-tumorigenic M1 and pro-tumorigenic M2, upon stimulation by different factors in the TME. This process is known as TAM polarization [18–20] (Fig. 2). The two types of macrophages possess obvious differences in terms of function, biomarkers, and metabolic activities. In general, M1-type TAMs, also called classically activated macrophages, are induced by T_H_1-type cytokines (e.g., TNF-α and IFN-γ) [21]. They can enhance anti-tumor immune response by producing pro-inflammatory cytokines (e.g., interleukin-1 (IL-1), IL-6, IL-12, IL-23, CXCL-10 and TNF-α), which help to recruit and activate immune cells to target the tumor. In addition, they can also mediate tumor cytotoxicity by producing reactive oxygen species or nitric oxide capable of inducing DNA damage and apoptosis [22]. Comparatively, M2-type TAMs, also known as alternatively activated macrophages, are typically induced by the T_H_2 cytokines IL-4/IL-13. They can secrete anti-inflammatory growth factors (e.g., VEGF and TGF-β), matrix metalloproteinases (MMPs) (e.g., MMP-2 and MMP-9), and other cytokines (e.g., IL-10, IL-13, and IL-4), playing a key role in promoting EMT, angiogenesis and immunosuppression [23]. Especially, their immunosuppressive effect ultimately contributes to tumor progression and unfavorable treatment outcomes. Furthermore, M1-type TAMs typically express CD80, CD86, CD64 and MHC II, while M2-type TAMs are characterized by CD163, CD206 and ARG1. Those biomarkers can be used for the classification and identification of TAM subsets. Notably, although the utilization of a binary polarization system is widespread in macrophage studies, emerging evidence suggests that TAMs usually exhibit a spectrum of phenotypes, expressing a combination of immunostimulatory and immunosuppressive markers in addition to the M1 and M2 polarization types [24]. Therefore, the conventional classification of macrophages into distinct M1 or M2 phenotypes may oversimplify their complexity. It is more accurate to describe macrophage polarization as a dynamic spectrum of phenotypes.

## Biological significance of TAM polarization in CRC progression and metastasis

Notably, both of M1- and M2-type TAMs can dynamically transform into each other with the changes in the TME. The polarization of TAMs is regulated by a wide range of cytokines, growth factors, chemokines, and other signals generated from tumor and stromal cells, playing a critical role in CRC progression and metastasis [25]. CCL2 is known as the predominant chemokine expressed by different tumor cells, and plays a pivotal role in the recruitment of immune cells, especially TAMs, through the CCL2/CCR2 axis [26]. Tu et al. found that CCL2/CCR2 interaction-mediated TAMs recruitment and M2 polarization contribute to the liver metastasis of CRC in the mouse model [27]. colony stimulating factor-1 (CSF-1) and IL-34, sharing a common receptor (CSF-1R), are potent macrophage recruiters and stimulating factors of M2-type TAM polarization. Gao et al. found that FOXO1 is a positive regulator of CSF-1, which can promote M2 macrophage polarization and further enhance cetuximab resistance in CRC [28]. In contrast, Nakanishi et al. demonstrated that the COX-2 inhibitor celecoxib altered the phenotype of TAMs from M2 to M1 in an IFN-γ-dependent manner, potentially reducing the progression of intestinal tumors, including CRC [29]. Ma et al. found that *miR-148a* can facilitate the transition of TAMs from the immune-suppressive M2 phenotype to the immune-promoting M1 phenotype in CRC [30]. Lai et al. found that lncRNA *NBR2* can regulate TAM polarization towards M1 phenotype, thereby suppressing the progression of CRC [31]. STAT3 is a pivotal transcription factor for TAM polarization. Inhibiting STAT3 signaling can promote macrophage polarization towards the M1 phenotype, which has anti-tumor properties [32]. Zhang et al. discovered that Cucurbitacin B could reduce the polarization of M2 macrophage by targeting the JAK2/STAT3 signaling pathway, thereby inhibiting CRC metastasis [33]. The flexibility of TAMs to shift between these phenotypes highlights their adaptability and responsiveness to microenvironmental factors. Therefore, exploring the molecular mechanisms underlying TAM polarization within the TME not only enhances our understanding of CRC pathogenesis, but also offers valuable insights for developing innovative cancer therapies.

## Multi-dimensional landscape of TAM-mediated cellular carcinogenesis in CRC metastasis

**Fig. 3:**
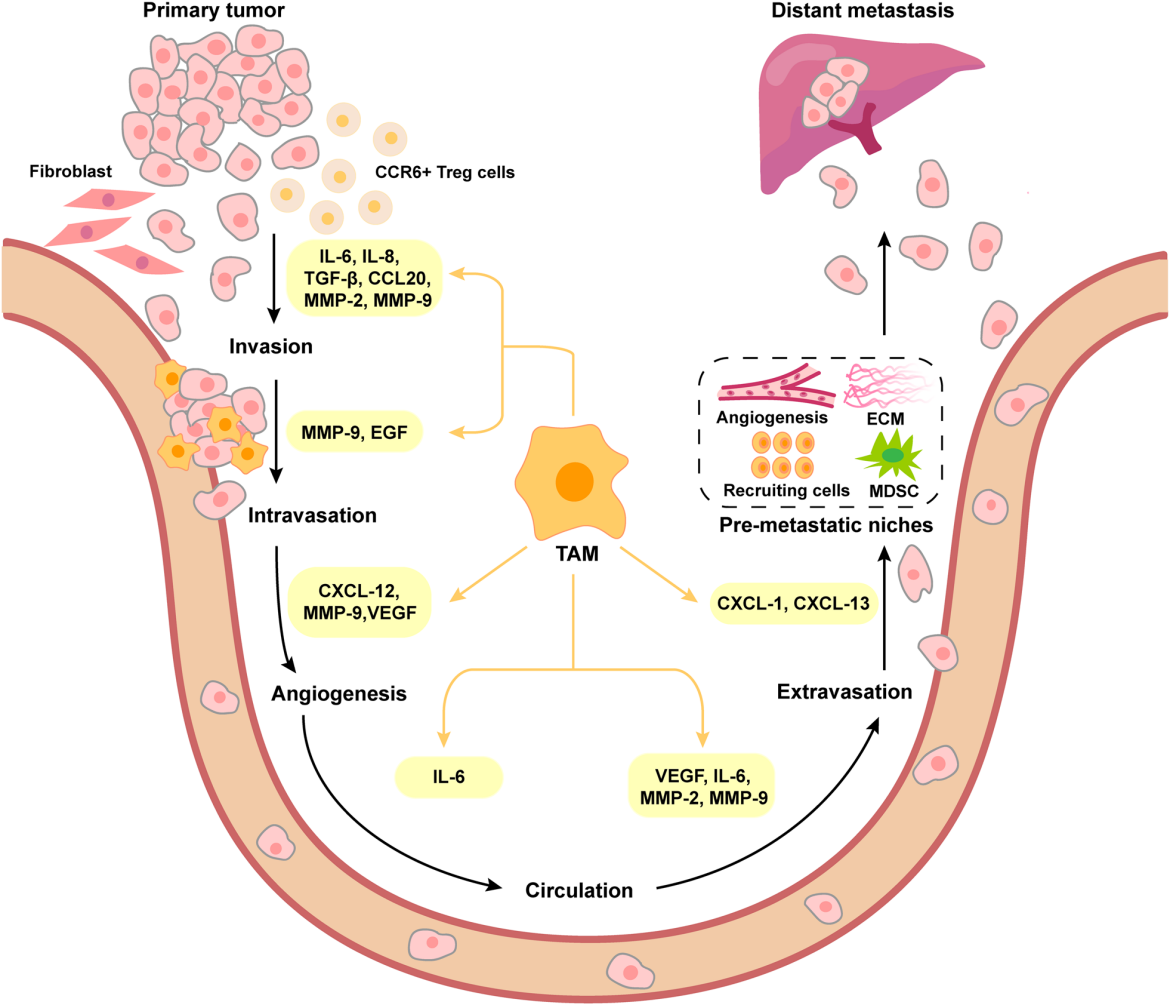
The main role of TAMs in the metastasis of CRC. TAMs contribute to multiple stages of CRC metastasis, including invasion, intravasation, angiogenesis, circulation, extravasation and PMN formation, by secreting different factors. *IL-6 *interleukin-6,* IL-8 *interleukin-8, *TGF-β *transforming growth factor-β, *CCL20 *C-C chemokine ligand 20, *MMP-20 *matrix metalloproteinase-20, *MMP-9 *matrix metalloproteinase-9, *EGF *epidermal growth factor, *CXCL-12 *C-X-C motif chemokine ligand 12, *CXCL-1 *C-X-C motif chemokine ligand 1,  *CXCL13 *C-X-C motif chemokine ligand 13, *VEGF *vascular endothelial growth factor, *ECM *extracellular matrix, *MDSCs *myeloid-derived suppressor cells

Metastasis formation is a multi-step cascade of events involving local invasion of tumor cells into surrounding tissues, intravasation of tumor cells into the bloodstream or lymphatic system, survival of tumor cells in the circulation, transportation of tumor cells to distant organs (typically liver and lung in CRC), extravasation and colonization of tumor cells to establish micro-metastases, and proliferation of tumor cells at the secondary sites. As an important immune cell type in TME, TAMs play an important role in regulating the process of CRC metastasis (Fig. 3).

### Activating the invasion of CRC cells

Metastasis begins with the invasion of tumor cells from the primary tumor into the surrounding normal stroma. The invasive capacity of tumor cells serves as the fundamental criterion for determining malignancy [34]. Recently, TAMs have emerged as pivotal players in promoting CRC invasion. For example, TAMs were found to regulate the invasive characteristics of CRC cells through the production of pro-inflammatory IL-6. This cytokine can activate the JAK2/STAT3 pathway, leading to enhanced expression of FoxQ1, thus promoting CRC invasion [10]. Xu et al. also demonstrated that TAM-derived IL-6 and IL-8 could favor the invasion of LoVo cells by activating the KCNN4/PRL-3 axis [35]. EMT process is characterized by the transformation of epithelial cells into mesenchymal cells, which plays a critical role in facilitating cancer cells to invade to surrounding tissues. Multiple TAMs-secreted cytokines (e.g., IL-8, IL-1β, TNF-α, and TGF-β) have been demonstrated to promote the EMT process in CRC [36, 37]. Cai et al. found that TAM-secreted TGF-β can contribute to the EMT process by mediating the Smad2,3–4/Snail signaling pathway, thereby promoting CRC invasion [38]. Moreover, TAMs may interact with neighboring cells within the TME, further contributing to CRC invasion. By using a CRC mouse model, Liu et al. found that TAMs recruited CCR6^+^ Treg cells to the tumor site by inducing CCL20 expression, promoting CRC development [39]. Zhang et al. demonstrated that cancer-associated fibroblasts (CAFs) could enhance TAM infiltration and M2-type polarization, consequently impairing the killing function of NK cells [40]. In addition, TAMs can secret multiple enzymes, such as MMP-2 and MMP-9, cathepsins, and fibrinolysin responsible for the degradation of extracellular matrix (ECM) components, thereby promote the local invasion of CRC cells [35, 41]. Therefore, TAMs could contribute to CRC invasion through the secretion of diverse factors and enzymes, and their interactions with neighboring cells within the TME.

### Inducing the angiogenesis of CRC cells

Angiogenesis plays a critical role in providing nutrients and oxygen to support the growth and progression of tumor. It has been shown that this process can be influenced by factors such as tumor grade and stage, the cellular composition of the TME (particularly the immune component), and the balance between pro-angiogenic and anti-angiogenic factors [42]. As an important type of immune cells in TME, TAMs can secrete chemokines that affect the formation of new blood vessels in CRC. It has been shown that the family of C-X-C chemokines possesses angiogenic or anti-angiogenic properties, depending on whether contain the ELR (Glu-Leu-Arg) motif. In general, the presence of this motif promotes angiogenesis, while its absence suppresses angiogenesis [43]. CXCL12 is known to facilitate angiogenesis, despite lacking the ELR motif. In a mouse model of inflammation-induced colon cancer, TAMs have been identified as a main driver of tumor neo-angiogenesis. This is achieved through their production of the pro-angiogenic factor CXCL12 [44]. Based on a tumor-on-a-chip platform, Bi et al. found that M1-type TAMs can inhibit angiogenesis and tumor growth by promoting the production of CXCL9, CXCL10, and CXCL11 in CRC [45]. Moreover, TAMs can release pro-angiogenic factors like MMPs and VEGF, which can degrade the ECM and promote the remodeling of blood vessels to support the growth and metastasis of CRC. Nocito et al. demonstrated that decreasing the expression of MMP-12 in tumor-infiltrating macrophages can reduce the level of angiostatin and thus regulating angiogenesis in a model of colon cancer allografts [46]. Xu et al. found that recruited TAMs can secrete MMP-9 and VEGF, providing a favorable environment for tumor angiogenesis and metastasis [47]. Overall, TAMs play a crucial role in promoting the angiogenesis of CRC cells, facilitating the growth and progression of CRC.

### Promoting the intravasation and extravasation of CRC cells

Intravasation and extravasation are crucial steps in the metastatic cascade. During intravasation, tumor cells detach from the primary tumor and infiltrate the endothelial wall of blood vessels, allowing them to enter the bloodstream and act as circulating tumor cells (CTCs) [48]. Comparatively, extravasation is the process by which CTCs exit the blood vessels at metastatic sites to initiate new colonies and establish secondary tumors [49]. CRC cells, like other cancer cells, rely on the intravasation and extravasation processes to successfully enter and exit the bloodstream.

Recent studies have shown that TAMs play a crucial role in promoting both intravasation and extravasation of CRC cells [50]. First, the production of growth factors by TAMs is one mechanism through which they can influence CRC cell behavior and facilitate these processes. It has been reported that TAMs-derived EGF can promote the invasion and motility of CRC cells [51], and the activation of EGFR are essential for maintaining tumor cell intravasation [52]. Similarly, Weis et al. demonstrated that the vascular endothelial growth factor (VEGF) can induce endothelial gaps and promote tumor cell extravasation by injecting VEGF-expressing CT26 colon cancer cells into mice [53]. Considering the ability to produce VEGF, TAMs may promote CRC extravasation by altering vascular permeability. Second, TAMs can produce various enzymes that further induce changes in the TME, making it more conducive for the intravasation and extravasation of CRC cells. Typically, TAMs can create a favorable microenvironment for CRC cell by secreting MMPs, which are enzymes responsible for degrading the basement membrane and modifying the composition of the ECM [54]. For example, CD155^+^ TAMs have been observed to induce the expression of MMP-9 [55], which can contribute to CRC intravasation. By using an orthotopic CRC model, Afik et al. demonstrated that monocyte-derived TAMs promote tumor development by reshaping the composition and structure of the ECM [56]. In addition, the crosstalk between TAMs and CRC cells may facilitate the intravasation and extravasation processes. Tumor cell-carrying macrophages could potentially interact with and influence the endothelial cell, which allow the entry of the tumor cell-macrophage complex into the bloodstream [50]. Therefore, the TAMs with pro-inflammatory and pro-angiogenic properties play a significant role in promoting the intravasation and extravasation of CRC cells, thereby favoring their metastatic spread.

### Enhancing the survival of CRC cells in the circulatory system

Metastasis is a very inefficient process, as only a small proportion of tumor cells survive in the bloodstream and further form metastatic sites in distant organs. Once tumor cells enter the bloodstream, they encounter new environmental challenges that may affect their survival [50]. Currently, there is emerging evidence suggesting that TAMs can enhance the survival of CRC cells in circulation by secreting cytokines and chemokines. For example, TAM-derived IL-6 can activate the JAK-STAT3 pathway, which promotes the survival and proliferation of tumor cells during the development of inflammation-related CRC [57]. In addition, TAMs-involved angiogenesis also provides nutrients and oxygen for CRC cells to survive in the circulation. Therefore, TAMs are an important factor affecting the survival of CRC cells in the circulatory system.

### Contributing to the formation of pre-metastatic niches (PMN) in CRC cells

Premetastatic niche (PMN) refers to the specialized microenvironment in distant organs that supports the establishment and growth of metastatic tumors. It is characterized by several features, including enhanced vascular permeability, remodeling of the ECM, angiogenesis, recruitment of bone marrow-derived cells, and immunosuppression [58]. In the metastatic cascade of CRC, in order for primary tumors to reach distant tissues with blood circulation, it is necessary to create PMN that can support tumor growth at the secondary site. TAMs play a critical role in the formation of PMN in CRC. For example, Wang et al. found that primary CRC cell-secreted VEGFA can trigger TAMs to produce CXCL1, which acts as a signal that attracts and recruits CXCR2-positive myeloid-derived suppressor cells (MDSCs). The accumulation of CXCR2-positive MDSCs in the liver facilitated the formation of PMN supporting the development of liver metastasis. Zhao et al. demonstrated that polarized M2 macrophages can secrete CXCL13, which in turn activates a positive feedback loop involving CXCL13/CXCR5/NFκB/p65/miR-934, leading to the induction of PMN formation in CRC cells [59]. Therefore, TAMs play an important role in the formation of PMN, which is closely related to the metastasis and growth of CRC cells.

## TAMs as therapeutic targets in metastatic CRC therapy

Since approximately half of the CRC patients eventually develop metastasis, the treatment strategies for metastatic CRC have long been a research focus in the medical field. The selection of suitable adjuvant treatments for metastatic CRC patients depends on various factors such as age, tumor stage, microsatellite instability status, presence of high-risk pathological features, and their overall performance status. The primary treatment for unresectable mCRC patients is systemic therapy, mainly including chemotherapy, immunotherapy, targeted therapy, and their combinations [60]. Especially, immunotherapy has rapidly emerged as a prominent and effective treatment approach for various solid tumors, including a subset of CRC. It has been reported that reducing the density or modulating the functions of TAMs is an effective approach for cancer immunotherapy. Moreover, inhibiting immunosuppressive activities of TAMs may improve the effect of chemotherapy and radiotherapy [61]. Therefore, targeting TAMs in CRC therapy holds great promise for improving prognostic outcomes of patients (Table 1) [62, 63].

**Table 1:** TAMs as promising targets for CRC treatment

Drug	Type	Target	Function	PMID
**Reprogramming TAMs**				
Anti-MARCO IgG	Monoclonal antibody	MARCO	Increasing population of M1 macrophages and decreasing population of M2 macrophages	27210762
Tasquinimod	S100A9 inhibitor	S100A9	Leading to the transformation of M2 macrophages into M1 macrophages	26673090
Actibind (T2 RNases)	Ribonuclease	N/A	Rebalancing the ratio of M1/M2 macrophages and recruiting CD8 + T cells	32197460
EZH2 inhibitor	Small-molecule inhibitors	STAT3	Leading to the transformation of M2 macrophages into M1 macrophages	35432300
**Inhibiting TAMs survival**				
Pexidartinib (PLX3397)	CSF1R inhibitor	CSF1R	Depleting M2 macrophages and increasing CD8^+^ T cell infiltration	36600555
Trifluridine/Tripiracil (FTD/TPI)	Combination drug of trifluridine and tipiracil	N/A	Depleting TAMs	31611243
Oxaliplatin	Platinum based chemotherapy drugs	N/A	Depleting TAMs	31611243
M2pep	M2 macrophage-targeting peptide	Macrophages	Reducing M2 macrophages	24046373
RG7155	Monoclonal antibody	CSF-1R	Depleting TAMs and increasing CD8^+^/CD4^+^ T cell ratio	24898549
Lenvatinib	Tyrosine kinase inhibitor	VEGF and FGF receptors	Reducing TAMs numbers	30811474
**Inhibiting TAMs recruitment**				
NT157	IGF-1R inhibitor	IGF-1R and STAT3	Inhibiting expression of CCL2 to inhibit recruitment of TAMs	26364612 34580284
AMG 820	CSF1R antibody	CSF1	blocking the CSF1-CSF1R signaling to inhibit recruitment of TAMs	28655795 35718267
PF-04136309	CCR2 inhibitor	CCR2	Inhibiting CCL2 binding to CCR2 to inhibit recruitment of TAMs	34729117 34580284

### Reprogramming TAMs

TAMs possess the ability to undergo dynamic switches in their phenotypes and functions in response to microenvironmental stimulus and signals. As mentioned above, M1-type TAMs play a crucial role in activating the adaptive immune response by producing immunostimulatory cytokines. Comparatively, M2-type TAMs can suppress adaptive immune responses by secreting anti-inflammatory factors. Therefore, strategies aimed at shifting the balance of macrophage polarization towards the M1 phenotype hold promise in the treatment of metastatic CRC.

Currently, several potential drugs have shown the ability to reprogram the phenotype of TAMs from M2 to M1, offering potential therapeutic benefits for CRC patients with metastasis. For example, Georgoudaki et al. demonstrated that treatment with anti-MARCO monoclonal antibody can reprogram TAMs into M1 phenotype and enhance their anti-tumor activity in the MC38 mouse model of colon carcinoma, showing potential in inhibiting the metastasis of CRC patients [9]. Tasquinimod, a quinoline-3-carboxamide analog, has shown potential as an anti-cancer drug for the treatment of prostate cancer as well as other types of solid tumors [64]. Olsson et al. observed that the anti-angiogenic and anti-metastatic effects of tasquinimod were closely associated with its ability to induce an early phenotypic switch of TAMs from M2-type to M1-type, as evidenced by a decreased expression of pro-angiogenic markers and an increased production of the anti-angiogenic IL-12 in the MC38-C215 colon carcinoma tumors [65]. Carvalho et al. discovered that M1-derived extracellular vesicles loaded with oxaliplatin, retinoic acid, and *Libidibia ferrea* can induce the transition of TAMs from M2 to M1 phenotype through the STAT3/NF-kB/AKT signaling pathway. This shift effectively inhibited the metastasis of CRC in mice [66]. Therefore, reprogramming TAMs represents a promising strategy for the treatment of metastatic CRC. It has the potential to enhance the anti-tumor immune response, and overcome immunosuppression in the TME.

### Depleting TAMs

TAMs in the TME primarily express phenotypes that inhibit adaptive immunity [61]. The abundance of TAMs within TME is closely associated with poor prognosis in patients with CRC and other types of malignancies, making TAM depletion a novel strategy of cancer immunotherapy. Zhu et al. found that the elevated expression of CSF1R in TAMs was linked to an unfavorable prognosis in CRC patients. Furthermore, they demonstrated that the CSF-1R inhibitor PLX3397 can deplete M2-type TAMs by blocking the CSF1R pathway, thereby inhibiting CRC growth and metastasis [67]. Using a mouse model, Qiao et al. observed that treatment with TMP195, a selective class IIa histone deacetylase inhibitor, led to a decrease in the population of F4/80^+^ TAMs. This reduction was found to effectively inhibit the growth and vascularization of colorectal liver metastasis [68]. In addition, trifluridine/tipiracil (FTD/TPI) is an innovative antimetabolite agent with immunogenic cell death induction activity for the treatment of chemorefractory CRC. Limagne et al. found that the combination of FTD/TPI with oxaliplatin can eliminate M2-type TAMs in CT26 tumor-bearing mice. Simultaneous administration of FTD/TPI and anti-PD-1 provides a promising treatment option for patients with metastatic CRC [69]. Therefore, these findings suggest that TAM depletion could be a valuable approach to improve overall therapeutic outcomes in CRC.

### Blocking TAMs recruitment

Tumors can produce cytokines, growth factors and chemokines to recruit TAMs to the tumor periphery, where they exert pro-tumor roles [70]. Hence, interference with those factor-related signaling using monoclonal antibodies or small molecule inhibitors could be a promising strategy to prevent TAM accumulation in the TME, and consequently enhance the effectiveness of CRC therapy.

As mentioned above, CCL2 is a crucial mediator of TAMs recruitment in the TME of CRC [27]. Sanchez-Lopez et al. investigated the effect of NT157, a unique inhibitor targeting IGF-1 receptor and STAT3, on the TME of CRC. Their findings revealed that NT157 can attenuate migratory and invasive characteristics of CRC cells, reducing their ability to form metastatic lesions in the liver. Additionally, they observed that NT157 not only reduced tumor burden but also suppressed the expression of various pro-tumorigenic chemokines (e.g. CCL2) and cytokines (e.g. IFN-γ and IL-1β), which are critical for TAM recruitment [71]. In addition, TAM recruitment and polarization are tightly regulated by the interaction of CSF-1 and its receptor CSF-1R. Indeed, Chen et al. found that CSF1R^+^ TAMs are the major components regulating PD-L1 expression within the immunosuppressive TME. Inhibition of tumor PD-L1 expression can reduce lung metastasis, providing a therapeutic strategy for advanced CRC patients by blocking the CSF1-CSF1R signaling pathway [72]. AMG 820 is a novel, fully human CSF1R antibody that is currently under investigation. It works by blocking the binding of CSF1 and IL34 ligands to the receptor CSF1R [73]. Razak et al. demonstrated the safety and efficacy of AMG 820 in combination with panitumumab for the treatment of advanced solid tumors including advanced CRC [74]. These results indicate that the inhibitor AMG 820 might hinder the recruitment of TAMs by decreasing the levels of CSF-1R in metastatic CRC. Therefore, by targeting the signals involved in TAM recruitment, it is possible to inhibit the infiltration of TAMs into the TME of CRC.

Although TAM-targeted therapies seemed to be powerful in preclinical models of metastatic CRC, it is still urgently needed to further validate their efficacy in clinical applications. First, as TAMs play important roles in tissue homeostasis and immune function, disrupting their function has unintended consequences on the host immune system, leading to potential side effects and toxicity. Second, the heterogeneity of TAMs and the lack of highly specific biomarkers make it challenging to target TAMs specifically without affecting other immune cell populations. Moreover, the complex and heterogeneous nature of TME could limit the penetration and efficacy of therapeutic agents targeting TAMs.

## Conclusions and perspectives

In the past decades, the advancement of treatment strategies has greatly improved the prognosis of CRC patients, but distant metastasis remains a challenge [34]. Moreover, the incidence rate of CRC among young people is increasing year by year [75]. Therefore, it is crucial to unravel the mechanisms of CRC metastasis and develop novel therapeutic strategies.

**Fig. 4:**
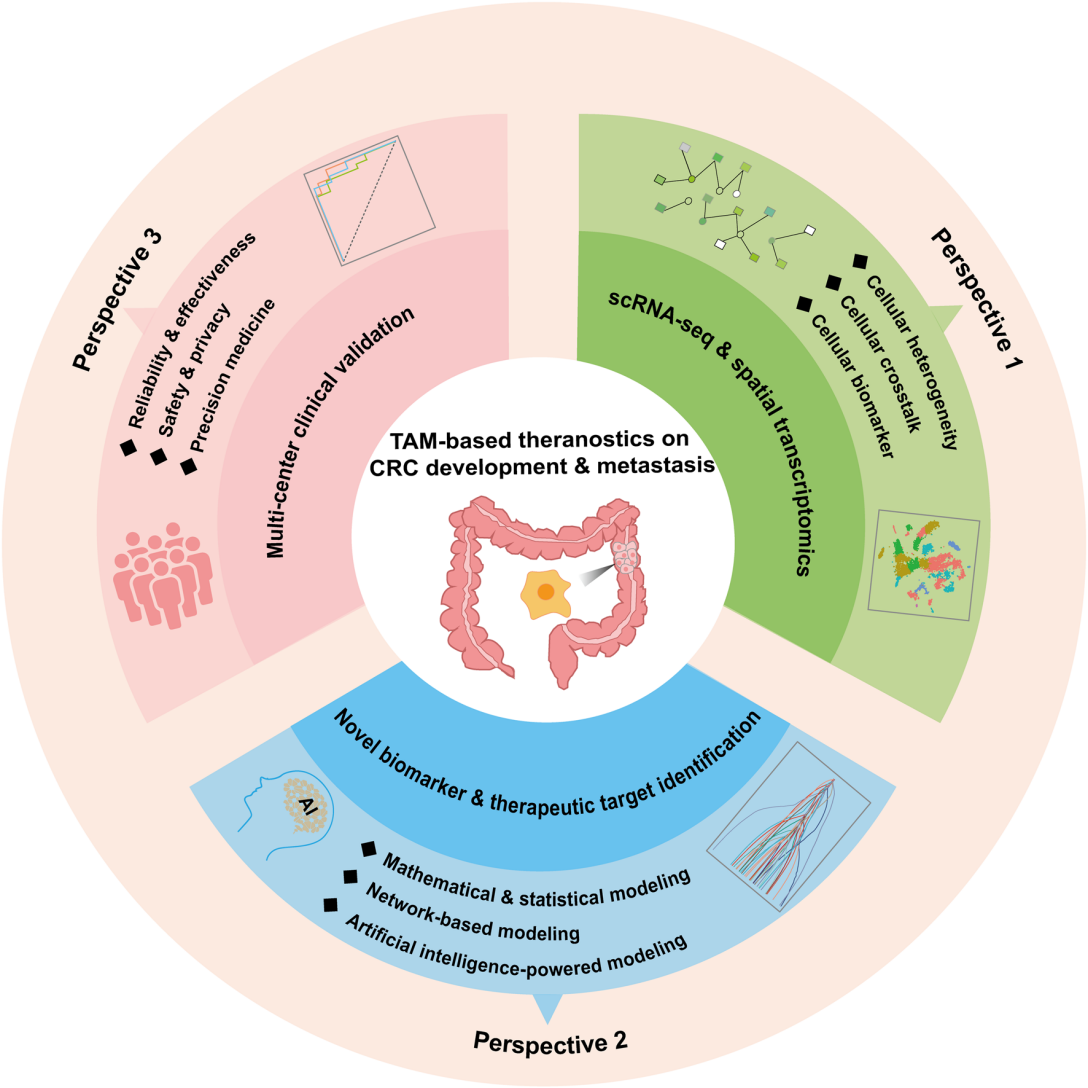
Translational perspectives of TAMs in CRC development and metastasis

Recently, the TME has gained increasing attention in cancer research. Various components of the TME including immune cells, endothelial cells, fibroblasts and ECM can interact with each other to form a favorable microenvironment for tumor growth and metastasis [76]. As the main immune component of TME, TAMs can be polarized into two distinct phenotypes: M1 and M2, and play a pivotal role in the metastatic cascade by multiple mechanisms, including promoting angiogenesis, suppressing immune responses, and remodeling the ECM. Given their critical role in tumor progression and metastasis, TAMs have emerged as promising targets for cancer therapy. For example, Williams et al. reported the potential of macrophage-targeted intervention strategies in significantly reducing the incidence and mortality of breast cancer in mouse models as well as early clinical trials [77]. Ma et al. found that reducing the expression level of *miR-182* in macrophages using an antagomiR inhibitor could inhibit M2 polarization of TAMs and suppresses breast cancer development [78]. In terms of CRC, several monoclonal antibodies or small molecule inhibitors have exhibited important roles in interfering with reprogramming, elimination and inhibition of TAMs, and hold potential for improving treatment outcomes and inhibiting tumor progression in CRC patients. As shown in Fig. 4, the opportunities and challenges of TAM-based theranostics on CRC development and metastasis were summarized from three perspectives.

### Perspective 1: Deciphering TAM heterogeneity and cellular interactions in CRC based on single-cell sequencing and spatial transcriptomics

Although there have been significant advancements in understanding the role of TAMs in CRC development and metastasis, there are still several issues requiring further clarification in TAMs studies. On the one hand, the heterogeneity and plasticity of TAMs make it insufficient to target any single factor associated with the recruitment, polarization and function of TAMs, posing significant challenges for both mechanistic studies and clinical translation of TAM-mediated therapies [79]. On the other hand, the presence of multiple cell types in the TME adds to the complexity of targeting TAMs effectively. TAMs can interact with cancer cells and other immune cells through direct cell-cell contact or by secreting factors such as cytokines, chemokines, growth factors, and extracellular matrix remodeling enzymes. In particular, these interactions are dynamic and complex in the TME, potentially impacting the response to TAM-related therapy.

Notably, single-cell RNA sequencing (scRNA-seq) and spatial transcriptome technologies are providing unprecedented insights into the heterogeneity, cellular crosstalk and spatial organization of TME during CRC metastasis and/or treatment. By performing combined scRNA-seq analyses on the TME in CRC and murine tumor models, Zhang et al. found that SPP1^+^ TAMs harbor pro-tumor and pro-metastasis roles, while C1QC^+^ TAMs have the potential to regulate anti-tumor T cell responses. The two TAM subsets exhibited differential sensitivity to CSF1R blockade, indicating the insufficiency of anti-CSF1R treatment to deplete all TAM populations [80]. Through single-cell and spatial analysis, Qi et al. demonstrated that SPP1^+^ macrophages may regulate FAP^+^ CAF through TGFB1, thereby promoting the secretion of MMPs and collagen to remodel the ECM, which further reduced the efficacy of PD-L1 therapy for CRC [81]. Moreover, the combination of scRNA-seq and spatial transcriptome technologies hold great promise for promoting the discovery of biomarkers by providing a deeper understanding of cellular diversity, spatial organization, and molecular changes associated with CRC. Therefore, understanding the heterogeneity and plasticity of TAMs, as well as their intricate interactions with various cell populations within the TME is crucial for the development of TAM-targeted therapies in CRC.

### Perspective 2: TAM knowledge-guided modeling and integrated screening of novel biomarkers and mechanisms for CRC precision medicine

TAM knowledge-guided modeling and integrated screening methods can help elucidate the underlying mechanisms of TAM and identify novel biomarkers, paving the way for the development of targeted therapies and personalized treatment strategies for CRC patients. First, mathematical and statistical models are commonly used to discover prognostic biomarkers associated with TAMs for CRC patients. By utilizing univariate Cox proportional hazards models, Yang et al. found that the CD163^+^/CD68^+^ ratio, which is an indicator of M2-type TAM polarization, at tumor invasive front serve as an independent prognostic factor for recurrence-free survival and overall survival in CRC patients. This finding highlights the significance of evaluating TAM polarization in assessing the prognosis of CRC patients [82]. Similarly, Malesci et al. discovered that high levels of TAMs, specifically in metastatic lymph nodes, can be used for identifying stage III CRC patients who would benefit from adjuvant therapy with 5-fluorouracil [83]. Second, network-based analysis, especially dynamic network analysis, can be used to capture changeable TAM-related signatures as candidate biomarkers or therapeutic targets in understanding and managing the complexities of CRC development and metastasis. By constructing a protein-protein interaction network, Cui et al. discovered that *PLAU* could serve as a potential TAM biomarker with predictive value for CRC invasion and metastasis [84]. Third, with the rapid progress of data-driven modeling and pattern recognition, artificial intelligence-powered approaches such as deep learning techniques are expected to be powerful tools for integrating TAM-based knowledge characterization to enhance the level of CRC precision medicine and holistic healthcare.

### Perspective 3: multi-center clinical validation of TAM-derived theranostics for CRC translational applications

It is imperative to conduct multicenter validation studies to evaluate the diagnostic accuracy, predictive value, and clinical outcomes of TAM-derived therapeutics for CRC patients. First, multi-center studies are essential for verifying the effectiveness and reliability of TAM-derived theranostics across diverse populations and clinical settings for CRC patients. By using transcriptomic data from 472 cases in the TCGA database and 964 cases in five GEO datasets, Ma et al. analyzed the expression of macrophage receptor CD163 in CRC patients. They found that the expression of CD163 is closely linked to the TME as well as tumor purity in CRC, indicating that it serves as a potential biomarker for predicting the prognosis of CRC patients [85]. Second, multicenter clinical validation should be conducted under a safe framework and the patient data privacy of patients should be considered and protected. Third, multi-center clinical validation could help accelerate the pace toward precision CRC medicine. In a study conducted by Hu et al., the heterogeneity of microsatellite instability-high (MSI-H) subtypes in CRC patients was analyzed. The findings suggested that PD-1/PD-L1 immunotherapy may not be suitable for all patients, and more clinical measures should be taken for selected CRC patients to improve their survival rate [86]. Therefore, the multi-center validation study is expected to provide strong support to evaluate the value of TAM in clinical practice for CRC patients.

In conclusion, this review discusses the origin, polarization, and functions of TAMs, and summarizes their involvement in the metastatic cascade of CRC. Importantly, TAMs hold promise as potential targets for CRC treatment. Although there are still challenges to overcome, the rapid advancements in biological technology offer hope that TAMs could serve as a pivotal area of breakthrough in future CRC therapies.

## Abbreviations

CRC: Colorectal cancer
TME: Tumor microenvironment
TAMs: Tumor-associated macrophages
EMT: Epithelial-mesenchymal transition
BMDMs: Bone marrow-derived macrophages
TRMs: Tissue-resident macrophages
CCL2: C-C chemokine ligand 2
CCL5: C-C chemokine ligand 5
CCL7: C-C chemokine ligand 7
CCL20: C-C chemokine ligand 20
CXCL8: C-X-C motif chemokine ligand 8
CXCL12: C-X-C motif chemokine ligand 12
GM-CSF: Granulocyte macrophage-colony stimulating factor
M-CSF: Macrophage-colony promoting factor
VEGF: Vascular endothelial growth factor
PDGF: Platelet derived growth factor
HSCs: Hematopoietic stem cells
GMPs: Granulocyte-macrophage progenitors
MDPs: Monocyte-dendritic cell progenitors
TNF-α: Tumor necrosis factor-α
IFN-α: Interferon-α
IFN-γ: Interferon-γ
IL-1: Interleukin-1
IL-6: Interleukin-6
IL-12: Interleukin-12
IL-23: Interleukin-23
CXCL-10: C-X-C motif chemokine ligand 10
TGF-β: Transforming growth factor-β
MMPs: Matrix metalloproteinases
MMP-2: Matrix metalloproteinase-2
MMP-9: Matrix metalloproteinase-9
IL-10: Interleukin-10
IL-13: Interleukin-13
IL-4: Interleukin-4
CCR2: C-C motif chemokine receptor 2
CSF-1: Colony stimulating factor-1
IL-34: Interleukin-34
CSF-1R: Colony stimulating factor-1 receptor
EGF: Epidermal growth factor
SMAR1: Scaffold/matrix attachment region-binding protein 1
IL-8: Interleukin-8
IL-1β: Interleukin-1β
CAFs: Cancer-associated fibroblasts
ECM: Extracellular matrix
CTCs: Circulating tumor cells
ELR: Glu-Leu-Arg
MDSCs: Myeloid-derived suppressor cells
FTD/TPI: Trifluridine/tipiracil
